# Deciphering the Link Between Diagnosis-Related Group Weight and Nursing Care Complexity in Hospitalized Children: An Observational Study

**DOI:** 10.3390/children12010103

**Published:** 2025-01-17

**Authors:** Manuele Cesare, Fabio D’Agostino, Emanuele Sebastiani, Nursing and Public Health Group, Gianfranco Damiani, Antonello Cocchieri

**Affiliations:** 1 Gemelli IRCCS University Hospital Foundation, 00168 Rome, Italy; 2 Department of Medicine, Saint Camillus International University of Health Sciences, 00131 Rome, Italy; fabio.dagostino@unicamillus.org; 3 Department of Biomedicine and Prevention, University of Rome Tor Vergata, 00133 Rome, Italy; sebastiani@agenas.it; 4 Catholic University of the Sacred Heart, 00168 Rome, Italy; nursingandpublichealthgroup@gmail.com; 5 Section of Hygiene, Woman and Child Health and Public Health, Gemelli IRCCS University Hospital Foundation, Catholic University of the Sacred Heart, 00168 Rome, Italy; gianfranco.damiani@policlinicogemelli.it (G.D.); antonello.cocchieri@policlinicogemelli.it (A.C.)

**Keywords:** standardized nursing terminologies, nursing complexity of care, medical complexity, diagnosis-related group, children

## Abstract

Background/Objectives: The increasing medical and nursing care complexity in hospitalized children represents a significant challenge for healthcare systems. However, the link between these two dimensions remains partially explored. This study aims to decipher the relationship between Diagnosis-Related Group (DRG) weight and nursing care complexity in hospitalized children and to identify the determinants of medical complexity. Methods: This retrospective study, conducted in an Italian university hospital, included children aged 2 to 11 years admitted to the hospital in 2022 with a minimum hospital stay of 2 days. Data were gathered from the Neonatal Pediatric Professional Assessment Instrument and the Hospital Discharge Register. DRG weight was used as an indicator of medical complexity, while the number of nursing diagnoses (NDs) documented in the first 24 h from hospital admission and the nursing actions (NAs) recorded during the patient’s hospital stay were used to measure nursing care complexity. Correlation analyses were conducted to explore the associations between DRG weight, NDs, and NAs. Stepwise regression was run to identify the key determinants of medical complexity across sociodemographic, clinical, organizational, and nursing variables. Results: Among 914 patients (mean age of 6.11 ± 2.90 years), the median DRG weight was 0.6982 (IQR: 0.5522). Patients had an average of 3.89 ± 2.83 NDs and a median of 17 NAs (IQR: 8). Significant correlations were found between NDs and NAs (r_s_ = 0.507; *p* < 0.001), as well as between DRG weight and the frequency of NDs (r_s_ = 0.232; *p* < 0.001) and NAs (r_s_ = 0.184; *p* < 0.001). Stepwise regression indicated that the number of NAs, surgical DRG, scheduled admissions, and ND frequency were significant determinants of DRG weight (R^2^ = 0.311; adjusted R^2^ = 0.308; *p* < 0.001). Conclusions: In children, DRG weight is also influenced by nursing care complexity, alongside clinical and organizational factors. An integrated approach is essential to enhance pediatric care and patient outcomes.

## 1. Introduction

Public health systems are increasingly challenged by the rising medical and nursing complexity of patient care, necessitating innovative solutions, advanced medical and nursing interventions, and integrated care models—particularly for children with acute and chronic conditions. Providing care for these patients often requires integrated and coordinated approaches to effectively understand and address their multidimensional needs [1]. Children’s care is often complicated by frequent transitions across multiple healthcare services, creating substantial care gaps due to fragmented coordination and service disjunctions. This patient population often experiences repeated hospitalizations, high health resource utilization, and elevated risks of medical errors [2,3]. The families of hospitalized children endure substantial stress and resource strain from fragmented care and multiple provider interactions. This combination of factors also impacts caregivers’ health and strains family resources, both financially and socially, underscoring the need for cohesive care models that better integrate medical and nursing complexities across settings to support timely, specialized, and accessible care [2,4,5,6].

In the literature, although no shared definition exists, medical complexity can be described as the intensity and resources required to care for a patient (e.g., presence of medical technologies, devices, and chronic conditions) [1]. This concept, historically quantified by Diagnosis-Related Group (DRG) weight [7], reflects the clinical acuity and resource intensity required for patient management [8] from a medical perspective (e.g., medical diagnoses and medical procedures). DRG weight is a standardized metric that assigns a numerical value to hospital cases based on the expected resource use, encompassing the severity of a patient’s condition and the complexity of required interventions. Today, DRG weight is widely used in healthcare systems to guide resource allocation, predict hospitalization costs, and facilitate care planning [9]. However, other key aspects of care, such as nursing care—which can also predict costs—are not considered in the DRG [10,11]. DRG weight reflects the standardized reimbursement hospitals receive for each case, proportionate to the resources required for patient care. Medical complexity is often simplified into components like DRG weight for ease of understanding and clarity [12]. However, its underlying determinants remain only partially understood, and ongoing research continues to deepen insights into this area [13]. To our knowledge, it is not known whether nursing care can be a determinant of medical complexity.

Nursing care complexity can be described by nursing diagnoses (NDs) and nursing actions (NAs) [14]. NDs represent the patient needs identified by nurses, which in some cases can be associated with medical conditions (e.g., the ND of anxiety before surgery). NAs encompass the interventions delivered in clinical practice to address these needs and the broader medical complexity.

Despite the acknowledged value of each form of complexity in contributing to a comprehensive understanding of children’s care, the interplay between medical and nursing complexities requires further investigation [15]. To date, only one study has investigated this relationship in pediatric patients [16]. However, it primarily used the number of chronic conditions as a proxy for medical complexity, rather than DRG weight. As the authors suggest, exploring alternative definitions of medical complexity, such as DRG weight, may offer clearer insights into how nursing care influences medical complexity [16].

The aim of this study is to analyze the relationship between medical complexity, as measured by DRG weight, and nursing care complexity, as measured by NDs and NAs, in hospitalized children. By examining these interrelations, this study seeks to identify the key determinants of medical complexity, thereby enhancing the understanding of factors that influence hospital resource utilization in children. Specifically, this study addresses two key questions: What is the relationship between medical complexity, as measured by DRG weight, and nursing care complexity, as measured by the number of NDs and NAs, in hospitalized children? Additionally, what key sociodemographic, clinical, nursing, and organizational factors are linked to increased levels of medical complexity in this pediatric population?

## 2. Materials and Methods

### 2.1. Study Design

This study utilized a retrospective observational design, analyzing data collected over a one-year period. This design builds on a previously published protocol by the authors, in which a structured approach was established for evaluating nursing and medical complexity in pediatric patients [14].

### 2.2. Setting, Participants, Inclusion and Exclusion Criteria

This research was conducted at an Italian university hospital, providing tertiary care services to pediatric patients. The study population included all children admitted in 2022 and hospitalized for all causes (acute or chronic conditions), aged from early to middle childhood (2 to 11 years), as defined by the National Institute of Child Health and Human Development’s age criteria [17]. The enrolled sample met the standard of a minimum hospital length of stay of 2 days, as a 1-day stay was considered a day hospital visit. Exclusion criteria included children admitted for day hospital or day surgery purposes and those whose parents or legal guardians did not provide informed consent for this study.

### 2.3. Data Sources and Collection Strategy

Data for this study were collected from two primary sources:

- Neonatal Pediatric Professional Assessment Instrument (PAI*ped*) [14]. First introduced in the clinical setting in 2016, this clinical nursing information system is specifically designed to help nurses better understand and manage the complexities of caring for children in hospital settings. Embedded into the electronic health record (EHR) of the major general hospital in Italy, PAI*ped* allows nurses to collect and organize information from the earliest hours of a child’s stay, covering various aspects of child health needs, including the documentation of standard NDs and NAs. PAI*ped* assists nurses in choosing the right nursing care plans (i.e., NDs and NAs) based on a child’s responses to medical diseases or life conditions. Using a validated clinical decision support system—specifically a scientifically validated algorithm known as the Nursing Assessment Form (NAF) with strong content validity [16]—PAI*ped* gives nurses suggestions tailored to the needs of each child, helping to ensure their care is as responsive and complete as possible. However, nurses have the flexibility to accept or adjust these recommendations, ensuring that every child’s unique situation is respected and thoroughly addressed. Data extracted from PAI*ped* included the number of NDs recorded within the first 24 h of admission, as well as the total number of NAs carried out throughout the patient’s hospitalization.
- Hospital Discharge Register (HDR). The HDR is a standardized tool—centralized at Ministry of Health—that captures key details about patients upon discharge, including demographics, medical diagnoses, and DRG weight. This information supports hospitals in resource planning and enables consistent data collection, which is essential for research and quality improvement [18]. Through HDR data, healthcare professionals can track patient trends across diverse populations, informing both clinical practice and policy development.

### 2.4. Variables

In this study, a range of variables was assessed to examine the relationship between DRG weight and nursing care complexity. Each variable was chosen to provide a comprehensive view of both medical and nursing demands, encompassing sociodemographic, medical, nursing, and organizational factors.

The information collected regarded the following:

- Sociodemographic and organizational data, including patient age and gender, modality of admission (scheduled or urgent from the emergency department), recovery setting (e.g., medical or surgical wards, ICU), and discharge disposition (e.g., home, inter-hospital patient transfer, voluntary, or died).
- Medical data, such as the primary medical diagnosis and comorbidities, recorded according to the International Classification of Diseases, 9th Revision, Clinical Modification (ICD-9-CM) system, to provide insight into the overall health status of each child. Additionally, the DRG category (medical or surgical) was included to distinguish cases based on the type of treatment needed, while DRG weight was used as a measure of medical complexity, serving as a standardized metric that classifies cases by clinical and resource-intensive needs [8,9].
- Nursing data. Nursing care complexity was assessed through NDs, which reflect the specific health needs and potential risks identified by nurses in response to the child’s clinical condition. For this study, the number of NDs recorded within the first 24 h from admission was used. NDs were recorded using the Clinical Care Classification (CCC) System [19], a globally recognized standardized nursing terminology validated for its effectiveness in pediatric care [16]. Similarly, NAs—also encoded within the CCC framework and representing the tasks performed by nurses to address each child’s identified health needs and risks through NDs—were quantified by calculating the total number of actions throughout the hospital stay. NAs refine nursing practice by assigning specific qualifiers, such as “assess”, “perform”, “teach”, or “manage”, ensuring the accurate documentation of the care process. Collectively, the number of NDs and NAs served as key indicators of the intensity and breadth of nursing care provided, reflecting the overall nursing care complexity [16].

### 2.5. Data Analysis

Data analysis was conducted to ensure a thorough examination of the relationships among variables. Descriptive statistics were calculated for all variables to provide a general overview of the study population, summarizing their characteristics. The normality of the variables was evaluated by examining skewness and kurtosis. Values exceeding an absolute skewness of 2 or kurtosis of 7 were considered indicative of non-normality [20]. Means, medians, and standard deviations were reported for continuous variables, while categorical variables were summarized as frequencies and percentages. Spearman’s correlation coefficients were used to assess the associations between DRG weight, the number of NDs, and the number of NAs. This preliminary analysis aimed to explore the strength and direction of the relationships among medical complexity, NDs, and NAs. A stepwise regression analysis was performed to identify the determinants of medical complexity. DRG weight was used as the dependent variable, while sociodemographic, medical, organizational, and nursing variables served as determinants. Dummy variables were included to code categorical data, allowing them to be included in the regression analysis as dichotomous determinants, facilitating the identification of variables that significantly contribute to medical complexity in children. Multicollinearity was evaluated using the variance inflation factor (VIF), with values below 5 indicating acceptable levels of collinearity among determinants [21]. All statistical analyses were two-sided, with a *p*-value of <0.05 set as the threshold for statistical significance. Data were analyzed using IBM SPSS Statistics^®^ version 29 for Mac OS (IBM Corp., Armonk, NY, USA).

### 2.6. Ethical Considerations

Approval for this study was obtained from the Catholic University of the Sacred Heart Ethical Committee (Approval No. 0012915/24). In line with ethical standards for retrospective research, informed consent was requested and obtained from parents or legal guardians for the use of data. The data were subsequently entered and anonymized in a dataset to protect patient confidentiality, minimizing risks to patients’ privacy. Additional consent was obtained from healthcare personnel responsible for clinical documentation to ensure the ethical use of professional contributions. This study adhered to the principles of good clinical practice and the Declaration of Helsinki, ensuring full compliance with patient data protection and privacy standards.

## 3. Results

### 3.1. Sociodemographic and Organizational Characteristics of the Sample

The study sample consisted of 914 children, with a mean age of 6.11 ± 2.90 years, the majority of whom were male (59.8%). Most children were admitted to medical wards (69.2%) through scheduled admissions (57.8%), and nearly all were discharged home (97.0%) (Table 1).

### 3.2. Clinical and Nursing Characteristics of the Participants

The most prevalent DRG categories were neurological and psychiatric, with “Seizure and Headache (Age 0–17)” being the most frequent (11.9%). Most patients were classified under medical DRGs (74.8%), with a median DRG weight of 0.6982 (IQR 0.5522) and a range from 0.2085 to 15.5111, reflecting substantial variability in case complexity. The most common primary medical diagnosis was “Autistic disorder, current or active state” (6.5%), with patients having an average of 1.90 ± 1.17 comorbidities.

Nursing complexity was described by an average of 3.89 ± 2.83 NDs per patient and a median of 17 NAs (IQR: 8). The most frequent ND was “Fall Risk” (82.6%), while the most prevalent NA was “Perform Individual Safety” (4.69%) (Table 2).

### 3.3. Relationship Between Medical Complexity and Nursing Complexity of Care

The Spearman correlation analyses revealed significant positive associations between DRG weight and the number of both NDs and NAs. Specifically, a moderate positive correlation was observed between NDs and NAs (ρ = 0.507; *p* < 0.001). Furthermore, DRG weight demonstrated a moderate positive correlation with the number of NDs (ρ = 0.232; *p* < 0.001) and a weaker positive correlation with the number of NAs (ρ = 0.184; *p* < 0.001).

### 3.4. Determinants of Medical Complexity Among the Sociodemographic, Organizational, Clinical, and Nursing Characteristics of the Sample

The stepwise regression model presented in Table 3 explored the determinants of medical complexity, with DRG weight as the dependent variable. In Model 1, the number of NAs significantly contributed to DRG weight (B = 0.045, *p* < 0.001) with an R^2^ of 0.184. Model 2 added DRG category as an additional variable, showing an increased R^2^ of 0.289; both the number of NAs (B = 0.041, *p* < 0.001) and surgical DRG category (B = −0.914, *p* < 0.001) were significant. Model 3 further incorporated the modality of admission, increasing R^2^ to 0.299, with all three variables (NAs, surgical DRG category, and scheduled modality of admission) showing significance (*p* < 0.001). Finally, Model 4 included the number of NDs as an additional predictor, achieving an R^2^ of 0.311, making it the best model among those tested. All determinants were significant contributors to DRG weight in this last model (*p* < 0.001). The variance inflation factor (VIF) values across all models ranged from 1.000 to 1.435, indicating low multicollinearity among the determinants. Age and gender were found to be non-significant across all models (Table 3).

## 4. Discussion

This study aimed to explore the relationship between medical complexity, as indicated by DRG weight, and nursing care complexity, measured by NDs and NAs, in hospitalized children. Additionally, this study sought to identify the determinants of DRG weight by examining a range of sociodemographic, medical, organizational, and nursing variables. This approach was intended to be used to uncover how these factors collectively or individually contribute to variations in DRG weight. Understanding these determinants can help healthcare providers and administrators optimize resource allocation, enhance care quality, and tailor interventions according to patient complexity [22].

The results revealed that DRG weight, a recognized measure of medical complexity [8,9], is significantly influenced by nursing care complexity, as well as specific medical and organizational factors. This finding underscores the interconnection of medical and nursing needs in children’s care and highlights the importance of incorporating both dimensions into resource planning and care optimization for this vulnerable population [23]. In particular, the correlation analyses showed moderate positive associations between DRG weight and both NDs and NAs. The relationship between DRG weight and NDs was stronger than that with NAs, which could be explained by the fact that NDs represent patient responses to health conditions. Therefore, the higher the patient’s DRG weight, the more patient responses are likely to be documented as NDs within the first 24 h from hospital admission. This aligns with the existing literature, which suggests that NDs provide insight into the patient’s overall health status and risk factors [14], elements that often correlate with higher clinical and resource needs in complex cases. The weaker correlation between DRG weight and NAs may reflect the variability in nursing activities, which, while integral, are tailored to specific patient needs and can vary in response to real-time clinical changes [14,24].

The stepwise regression analysis further supported these findings, with a high number of NAs, surgical DRG category, scheduled admission modality, and a high frequency of NDs emerging as significant determinants of DRG weight. Among these, the presence of a surgical DRG showed the strongest contribution to DRG weight, followed closely by a scheduled modality of admission and the number of NDs and NAs. This reinforces the idea that higher nursing care requirements, especially in scheduled pediatric cases requiring surgical intervention, are linked to increased medical complexity. Assessing nursing demands by specialty and procedure type may be essential for early insights into medical complexity in children. By using the frequency of NDs identified during the first 24 h from hospital admission and the number of NAs as primary indicators, this approach could facilitate the anticipation of resource needs and optimize care for children across various surgical contexts. Furthermore, given the variability in nursing complexity across different contexts and DRGs [25], focused nursing assessments by surgical cases and specialty could enhance resource allocation, staffing, and billing accuracy. Future research should further explore how nursing complexity differs across pediatric surgical specialties and procedures, aligning with the literature on medical complexity [26,27]. Additionally, studies should consider the distribution of NDs and NAs across specific diseases and disorders, providing a clearer understanding of medical complexity in relation to diverse clinical profiles.

Scheduled admissions were also associated with higher DRG weights, likely due to the medical complexity and planning required for elective procedures, which often include complex surgeries [28]. In pediatric care, these planned admissions necessitate detailed preparation and coordination to address children’s specific developmental needs, frequently involving multi-specialty teams to ensure comprehensive care [29]. This was confirmed by our sample, predominantly consisting of admissions with a surgical DRG, underscoring the medical complexity and specialized planning required in these cases.

Finally, NDs demonstrated their predictive value for medical complexity. NDs identified patient needs early in hospitalization, reflecting a range of clinical challenges and enabling nurses to systematically assess and prioritize care [30]. Common NDs, such as Fall Risk, Infection Risk, and Acute Pain, highlighted crucial areas of vulnerability in this population. The link between NDs and DRG weight underscored the critical role of NDs in providing structured and standardized data that not only inform individualized care but also, when compared with medical and organizational data, support resource planning, allocation, and comprehensive, responsive care for children in hospital settings.

The lack of significant associations between demographic factors like age and gender with DRG weight suggests that, in pediatric populations, medical and nursing complexities may be primarily driven by clinical and care-related factors rather than demographics alone. Further research could explore how specific clinical and nursing variables contribute to medical complexity across diverse pediatric subgroups, potentially enhancing precision in complexity prediction.

These insights have critical implications for children’s hospital care. The significant role of nursing complexity metrics in predicting DRG weight suggests that integrating standardized nursing terminologies (SNTs), such as NDs and NAs, with medical metrics in EHRs could improve the accuracy of resource planning and patient risk stratification. This integration could enhance hospital administrators’ ability to allocate resources efficiently, potentially reducing care gaps, supporting comprehensive, patient-centered care models, and ultimately improving healthcare outcomes [31,32].

The findings also highlight the benefits of using SNTs, such as the CCC System, for documenting NDs and NAs. SNTs support consistent and comparable data collection [33], enabling a more accurate assessment of nursing care complexity and facilitating the development of predictive models [31]. This standardization can contribute to an integrated approach in which nursing and medical metrics work synergistically to optimize patient care and outcome prediction in pediatric hospital settings.

### Limitations

Several limitations should be considered when interpreting our results. First, this study’s retrospective design and reliance on existing EHRs’ data may introduce biases or limit control over data accuracy. Furthermore, this study’s focus on a single Italian hospital may affect the generalizability of the findings to other pediatric populations or healthcare systems. This single-center approach may reflect specific institutional practices that differ from those in other settings. Another limitation is this study’s operational definitions of medical and nursing complexity, which, while recognized, could impact the results by constraining the range of complexity factors considered. Future research should work to validate these findings in diverse pediatric populations and healthcare settings and consider alternative or expanded definitions of medical and nursing complexities to capture a fuller understanding of the interconnected nature of these factors.

## 5. Conclusions

This study demonstrated that DRG weight, an indicator of medical complexity, was significantly related to nursing care complexity, as measured by NDs and NAs, along with clinical and organizational factors. These findings underscore the importance of an integrated approach to assessing medical and nursing complexities in pediatric hospital settings. By considering both medical and nursing data, healthcare providers can enhance resource allocation, improve care planning, and ultimately optimize outcomes for pediatric patients. Future studies should build upon these insights to develop predictive models that incorporate both medical and nursing metrics, enabling more tailored, effective interventions for children with high-complexity care needs.

## Figures and Tables

**Table 1:** Sociodemographic and organizational characteristics of the sample (N = 914).

Variables	Descriptive Statistics	
**Age** (years) (mean (SD); range)	6.11 (2.90)	2–11
**Gender** (n; %)		
Male	547	59.8
Female	367	40.2
**Modality of admission** (n; %)		
Scheduled	528	57.8
Urgent (from ED)	386	42.2
**Recovery setting** (n; %)		
Medical Wards	632	69.2
Surgical Wards	189	20.7
ICU	93	10.1
**Discharge disposition** (n; %)		
Home	887	97.0
Inter-hospital patient transfer	19	2.1
Voluntary	6	0.7
Died	2	0.2

Legend: SD, standard deviation; ED, emergency department; ICU, intensive care unit.

**Table 2:** Clinical and nursing characteristics of the sample (N = 914).

Variables	Descriptive Statistics	
**Ten most prevalent DRGs** (n; %)		
Seizure and Headache (Age 0–17)	121	11.9
Organic Disturbances and Intellectual Disability	94	9.3
Other Disorders of Nervous System Without Complication or Comorbidities	82	8.1
Childhood Mental Disorders	65	6.4
Craniotomy (Age 0–17)	54	5.3
Degenerative Nervous System Disorders	32	3.2
Viral Illness and Fever of Unknown Origin (Age 0–17)	29	2.9
Bronchitis and Asthma (Age 0–17)	26	2.6
Appendectomy Without Complicated Principal Diagnosis Without Complications	24	2.4
**DRG category** (n; %)		
Medical	684	74.8
Surgical	230	25.2
**DRG weight** (median, (IQR); range)	0.6982 (0.5522)	0.2085–15.5111
**Five most prevalent medical diagnosis** (ICD-9-CM) (n; %)		
Autistic disorder, current or active state	59	6.5
Unspecified delay in development	33	3.6
Other specified congenital anomalies of the brain	27	3.0
Complex febrile convulsions	23	2.5
Mild intellectual disabilities	20	2.2
**Comorbidities** (mean (SD); range)	1.90 (1.17)	1–7
**Number of NDs** (N = 3.558) (mean (SD); range)	3.89 (2.83)	1–14
**Five most prevalent NDs (CCC)** (n; %)		
Fall Risk	755	82.6
Infection Risk	566	61.9
Acute Pain	419	45.8
Sleep Pattern Disturbance	314	34.4
Injury Risk	188	20.6
**Number of NAs** (N = 18.049) (median, (IQR); range)	17.00 (8)	6–153
**Five most prevalent NAs (CCC)** (n; %)		
Perform Individual Safety	847	4.69
Assess Sleep Pattern Control	831	4.60
Perform Physician Contact	783	4.34
Assess Nutrition Care	781	4.33
Perform Counseling Service	723	4.01

Legend: DRG, diagnosis-related group; ICD-9-CM, International Classification of Diseases, 9th Revision, Clinical Modification; CCC, Clinical Care Classification System 2.5; NDs, nursing diagnoses; NAs, nursing actions.

**Table 3:** Determinants of medical complexity among the sociodemographic, organizational, clinical, and nursing characteristics of the sample (N = 914).

Model	Variables	*B*	95% CI		*SE*	β	*p*-Value	VIF	R^2^	Adjusted R^2^
#1	Intercept	0.094	−0.047	0.235	0.072	/	<0.191	/	0.184	0.183
	Number of NAs	0.045	0.039	0.051	0.003	0.429	<0.001	1.000		
#2	Intercept	0.855	0.671	1.038	0.093	/	<0.001	/	0.291	0.289
	Number of NAs	0.041	0.035	0.047	0.003	0.392	<0.001	1.013		
	DRG category ^a^	−0.914	−1.068	−0.761	0.078	−0.328	<0.001	1.013		
#3	Intercept	0.854	0.672	1.036	0.093	/	<0.001	/	0.302	0.299
	Number of NAs	0.046	0.040	0.052	0.003	0.438	<0.001	1.200		
	DRG category ^a^	−0.882	−1.035	−0.729	0.078	−0.317	<0.001	1.025		
	Modality of admission ^b^	−0.280	−0.425	−0.135	0.074	−0.114	<0.001	1.188		
#4	Intercept	0.757	0.569	0.946	0.096	/	<0.001	/	0.311	0.308
	Number of NAs	0.041	0.034	0.048	0.003	0.390	<0.001	1.435		
	DRG category ^a^	−0.847	−1.000	−0.693	0.078	−0.304	<0.001	1.042		
	Modality of admission ^b^	−0.339	−0.486	−0.191	0.075	−0.138	<0.001	1.249		
	Number of NDs	0.050	0.022	0.077	0.014	0.116	<0.001	1.416		

Dependent variable: DRG weight. ^a^ 1 = medical; ^b^ 1 = urgent. Note: Age and gender not significant in all models. Legend: CI, confidence interval; *SE*, standard error; VIF, variance inflation factor; NAs, nursing actions; DRG, diagnosis-related group; NDs, nursing diagnoses.

## Data Availability

The data presented in this study are available upon request from the corresponding author due to privacy and ethical reasons.
